# Hyperparameter Optimization with Genetic Algorithms and XGBoost: A Step Forward in Smart Grid Fraud Detection

**DOI:** 10.3390/s24041230

**Published:** 2024-02-15

**Authors:** Adil Mehdary, Abdellah Chehri, Abdeslam Jakimi, Rachid Saadane

**Affiliations:** 1 LaGes, Hassania School of Public Works, Casablanca 20000, Morocco; mehdary.adil@ehtp.ac.ma (A.M.); saadane@ehtp.ac.ma (R.S.); 2 Department of Mathematics and Computer Science, Royal Military College of Canada, Kingston, ON K7K 7B4, Canada; 3 GL-ISI Team, Faculty of Science and Technology Errachidia, Moulay Ismail University, Meknes 50050, Morocco; ajakimi@yahoo.fr

**Keywords:** genetic algorithms, XGBoost, hyperparameter optimization, fraud detection, smart grids, SGCC dataset, electricity theft, metaheuristic algorithms

## Abstract

This study provides a comprehensive analysis of the combination of Genetic Algorithms (GA) and XGBoost, a well-known machine-learning model. The primary emphasis lies in hyperparameter optimization for fraud detection in smart grid applications. The empirical findings demonstrate a noteworthy enhancement in the model’s performance metrics following optimization, particularly emphasizing a substantial increase in accuracy from 0.82 to 0.978. The precision, recall, and AUROC metrics demonstrate a clear improvement, indicating the effectiveness of optimizing the XGBoost model for fraud detection. The findings from our study significantly contribute to the expanding field of smart grid fraud detection. These results emphasize the potential uses of advanced metaheuristic algorithms to optimize complex machine-learning models. This work showcases significant progress in enhancing the accuracy and efficiency of fraud detection systems in smart grids.

## 1. Introduction

Electricity theft is a widespread problem that presents considerable challenges to the stability and economic sustainability of energy distribution networks across the globe. Electricity theft, including illegal connections, meter tampering, and bypassing, has significant financial implications for utility companies. Additionally, it indirectly affects consumers by causing increased tariffs and undermining the reliability of energy distribution systems [1].

The emergence of smart grid technology has sparked a significant change in the way electricity theft detection is approached. Conventional approaches that rely on manual inspections and basic anomaly detection have shown to need to be revised when dealing with advanced theft techniques. As a result, there has been an increasing focus on utilizing sophisticated data-driven methods, specifically machine learning algorithms, to address this problem effectively [2].

Transformer-based models have become increasingly popular in deep learning because of their exceptional capacity to process sequential data, vision, and recognize patterns [3]. Initially developed for natural language processing tasks, these models have shown impressive flexibility in detecting intricate consumption patterns and anomalies that may indicate electricity theft [4]. Through the analysis of extensive datasets from smart meters and grid sensors, Transformers can identify anomalies that traditional detection methods often miss.

Nevertheless, the utilization of these cutting-edge technologies does come with its fair share of obstacles. Concerns surrounding data privacy, the importance of extensive and varied datasets for model training, and the ability to understand machine learning decisions are crucial factors to consider [5]. In addition, it is crucial to carefully consider the ethical implications of surveillance and data utilization in electricity theft detection to ensure compliance with legal standards and societal norms [6].

As we move forward, combining Transformer models with smart grid data offers a promising opportunity to improve the precision and effectiveness of identifying electricity theft. This integration not only helps utility companies protect their revenues but also enhances the overall sustainability and reliability of power distribution networks [7].

The area of electricity theft detection (ETD) in smart grids is experiencing rapid advancements, fueled by the incorporation of advanced machine learning (ML) and deep learning (DL) techniques. This comparative table offers a concise summary of various influential research papers that have made significant contributions to this field. Every paper undergoes a thorough analysis to showcase its distinct methodology, focal points, key findings, and broader implications.

The comparison also includes the findings of these studies and considers future research directions. Table 1 provides a summary of the work that is pertinent to the detection of electricity theft. Through an analysis of various approaches, including a combination of deep learning models and creative implementations of traditional ML algorithms, the table provides valuable insights into ETD research’s current state and potential future direction. This analysis provides valuable insights into current trends and breakthroughs in the field while highlighting the upcoming challenges and opportunities for further progress.

Analyzing the table with a focus on the methodologies, focal points, key findings, implications, results, and future perspectives, we gain a comprehensive understanding of the current trends and future directions in electricity theft detection (ETD) research.

**Methodological Diversity and Advancements:** The methodologies adopted in these studies show a prominent trend toward the integration of deep learning (DL) and traditional machine learning (ML) approaches for ETD. For instance, the hybrid LSTM–UNet–Adaboost model in the Energies 2020 paper [9] combines deep learning with ensemble learning techniques. Similarly, the CNN-Transformer model discussed in Sensors 2023 [12] illustrates the effectiveness of combining convolutional neural networks with transformer networks for feature extraction. These advanced strategies enable efficient pattern recognition in complex datasets. Furthermore, the use of class balancing techniques, such as Adasyn and IQMOT, specifically targets the issue of data imbalance, a common challenge in electricity consumption datasets.

**Focal Point—Electricity Theft Detection (ETD):** The primary focus across these studies is ETD, reflecting its significant economic impact due to revenue losses for electric companies. Each research paper approaches a specific aspect of ETD, ranging from identifying illegal patterns in electricity consumption to forecasting demand and detecting non-technical losses (NTLs).

The authors in [8] focus on detecting electricity theft by applying supervised learning techniques to smart meter data. The study highlights the significance of utilizing smart meter data to identify and prevent fraudulent activities associated with electricity consumption. The results indicate that supervised learning techniques are successful in identifying patterns that indicate electricity theft.

The authors in [9] propose a methodology that combines deep learning and ensemble learning to prevent electricity theft in smart grids. This work highlights the effectiveness of their approach in identifying and mitigating instances of electricity theft through a combination of deep learning models and ensemble techniques.

The authors in [10] investigate electricity demand forecasting using artificial intelligence techniques, focusing on Gokceada Island as a case study. While this study does not directly address electricity theft, it underscores the importance of accurate forecasting methods in managing energy resources effectively.

The authors in [11] propose a novel method based on time-series transformation and machine learning for detecting non-technical loss (NTL) fraud in utility companies. The study emphasizes the significance of addressing NTL fraud using advanced machine-learning techniques.

The authors in [12] present a hybrid CNN–Transformer network for detecting electricity theft in smart grids. Their approach combines convolutional neural networks (CNNs) and Transformer architectures to enhance the accuracy of electricity theft detection.

The authors in [13] propose a hybrid deep learning-based model for detecting electricity losses using big data in power systems. Their study underscores the importance of leveraging big data analytics and deep learning techniques for the accurate detection of electricity losses.

**Key Findings and Implications:** A consistent outcome across these studies is the enhanced detection rates and accuracy in identifying electricity theft. The high accuracy, as seen in the Mathematics 2022 study [11], which achieved a 98% accuracy rate, demonstrates the effectiveness of these modern analytical methods. The implications of these findings are substantial for utility companies, offering more reliable and efficient ways to mitigate electricity theft and manage electricity demand.

**Results—High Accuracy and Efficiency:** The results from these studies emphasize high accuracy and efficiency in detection. Notable achievements include significant Precision-Recall (PR-AUC) and Receiver Operating Characteristic (ROC-AUC) scores. The Sustainability 2022 paper [13], for instance, achieved a PR-AUC of 0.985 and an ROC-AUC of 0.987, indicative of a high degree of model reliability and accuracy.

**Future Perspectives—Optimization and Wider Application:** The future research directions suggest a need for further refinement of these models for greater efficiency and adaptability to different electricity grid systems. There is a clear need for ongoing research to adapt to evolving electricity theft techniques and integrate these models into real-world applications across various geographic regions.

In conclusion, the table reflects a significant shift towards sophisticated, hybrid analytical models in the field of ETD. These studies contribute to a deeper understanding of electricity consumption patterns and theft detection, offering effective tools for utility companies. The high efficacy demonstrated in these studies indicates a promising future, but there remains a need for continuous refinement and adaptation to keep up with the evolving nature of electricity theft and the expanding nature of power grid infrastructures.

This paper explores the integration of Genetic Algorithms with XGBoost for fraud detection in the State Grid Corporation of China (SGCC) dataset, which presents exciting opportunities for future research.

This study focuses on the task of fine-tuning intricate machine-learning models within the domain of electricity theft, a pressing concern for power utilities. We carefully fine-tune important hyperparameters, including learning_rate, n_estimators, max_depth, min_child_weight, gamma, subsample, colsample_bytree, reg_alpha, reg_lambda, scale_pos_weight, and objective, using GA. We then assess how these adjustments affect the performance of the model.

Firstly, utilizing this optimized model on diverse datasets from different regions can offer valuable insights into its adaptability and generalizability. This step is essential for gaining a comprehensive understanding of the model’s effectiveness in a wide range of real-world scenarios. Additionally, there is an opportunity to enhance detection capabilities by integrating XGBoost with other machine learning techniques, such as deep learning approaches. These hybrid models have the potential to provide more sophisticated and reliable solutions for intricate and ever-changing fraud detection problems.

## 2. Sensors + Integrating New Technologies + Electrical Grids = Smart Grid

The public power grid has been a cornerstone of the energy industry for almost a century, maintaining its infrastructure and operational principles. Nevertheless, the world has undergone significant transformations. There has been a rise in consumption for various reasons, such as the expanding population and the growing energy demand. Additionally, the introduction of advanced technologies like electric cars has further raised the issue of the need for energy. Furthermore, electricity theft, fraud, and uncontrolled utilization of energy resources have also contributed to this increase.

Throughout history, power grids have been equipped with advanced software to facilitate their communication, supervision, and control. However, these technologies are typically reserved for the transmission network, playing a crucial role in enhancing the system’s overall safety.

Similarly, the professional world quickly embraced the Internet for its applications in electrical networks. This was mainly conducted to ensure all parties in the energy market had access to fair and up-to-date information about the available power transfer capacity, also known as Available Transmission Capability (ATC). The Internet’s potential extends far beyond this application and continues to be envisioned for various areas of the power grid. These areas encompass web-based services, applications that do not necessitate real-time control, observation, and monitoring in situations where the importance of the information has not been established, and more.

The recent advancements in ICTs have provided unprecedented solutions for electricity networks, surpassing what was previously thought possible. Take, for instance, the potential of incorporating metering and energy management devices on customers’ premises. These sensors and actuators enable two-way communication with network players and possess onboard intelligence. This transformative capability is reshaping the outlook of these networks. The connection between the consumer and the system, whether through an energy supplier, aggregator, commercial broker, or distributor, can be established using different communication methods. However, this has a significant influence on the power system.

The network relies on a combination of sensors, actuators, embedded software, and communication systems. These include components, control centers, and various technologies such as PLC (Power Line Communications), fiber optics, 4G, 5G, Wi-fi, and more. These functions are particularly intriguing.

For the network’s observability, supervision, and control concerning production and consumption. This includes the use of intelligent sensors and their management, as well as the transmission and processing of a growing amount of data. It also involves the development of driver assistance software that incorporates real-time safety features, including advanced telecontrol at the distribution network level.

### 2.1. Smart Meters

The smart meter comes in different variations, including bidirectional broadband communication, with or without software for consumption control and service offerings. It utilizes various communication media to enhance its intelligence. Smart meters allow operators to effortlessly gather consumption data, enabling them to identify usage peaks, regulate consumption, and implement effective billing strategies. For consumers, smart meters provide the ability to measure and access information about their electricity consumption, allowing them to better manage and reduce usage and costs. Thanks to the advanced communication capabilities between the operator and the consumer, smart meters offer a range of functionalities that can be categorized into different sections:

Measurement Module: Utilizing an automated meter reading (AMR) system, several research studies focus on real-time consumption measurement and time-of-use analysis. Other works also investigated the performance of the network to ensure optimal functionality.

Communication Module: The role of communication systems is to relay collected data to consumers and providers effectively, carrying out control commands. Time-of-use billing control and pricing policies are important considerations for effective billing.

Control Module: Optimizing resource distribution, ensuring stable voltage levels, and monitoring system performance.

Timing Module: Achieving time synchronization between multiple meters using a common time source.

Login Module: Enabling authentication for authorized individuals to gain access.

### 2.2. A Phasor Measurement Unit (PMU)

PMUs, also known as Phasor Measurement Units, are utilized to assess and analyze electrical power systems. Electrical signals on a synchronized network can be accurately measured using PMUs. These measurements are taken at the same time from a synchronized source, providing immediate readings of voltages and currents. PMUs are used for advanced monitoring applications, specifically for assessing network conditions in terms of control and protection.

### 2.3. Sensors and Actuators Devices

Innovative sensors are installed at the customer’s location, either connected to the smart meter or utilizing advanced transmission technology. These sensors are used for security and energy management purposes, as well as for energy services. Detection stations utilize sensors to facilitate monitoring. Sensors receive analog impulses and convert them into digital values that can be easily interpreted. The data are processed using centralized processing units to derive meaningful values for system evaluation. The intelligence related to the various aspects of domestic or industrial consumption, with a focus on energy efficiency and network security. A prime example of this is a smart load-shedding device that is connected to household appliances and operates based on changes in frequency or voltage.

### 2.4. Data Storage and Vehicle-to-Grid (V2G)

Energy storage systems include a range of choices, such as flow batteries, ultracapacitors, flywheels, pumped hydro, superconducting magnetic energy storage, and compressed air. Conversely, vehicles will be equipped with rechargeable batteries to provide electricity to operators’ stations when there is a strong demand. Vehicles can either be directly connected to operators or accessed via parking lots.

## 3. Methodology

### 3.1. XGBoost

XGBoost, also known as eXtreme Gradient Boosting, is considered a highly advanced algorithm in the field of Gradient Boosting. This technique, originally implemented in C++, has become widely popular because it is available in multiple programming languages essential for Machine Learning, such as Python, R, and Julia [14].

Gradient Boosting is a Boosting algorithm that combines multiple “weak learners” to create a powerful “strong learner”. This process involves the integration of multiple algorithms with moderate performance, resulting in a combined model that is more efficient and satisfactory. The coordination of less capable individuals into more capable individuals occurs through a series of iterations, with each one estimating a particular variable of interest.

In regression scenarios, the process involves estimating outputs using model 1 and using the residuals of this model as the target variable for model 2, and so on.

In the process of classification, individuals are assigned an initial weight, which is adjusted based on the accuracy of the model’s predictions. These weights then play a role in the subsequent estimation using the model. Tree algorithms are often used as weak learners in Gradient Boosting.

The standout feature of XGBoost is its exceptional computational optimization, which enables lightning-fast calculations. This algorithm efficiently handles data in compressed blocks, speeding up the sorting process and allowing for parallel processing. In addition to its efficient implementation and superior performance, XGBoost stands out for its wide range of hyperparameters. This diversity gives practitioners full control over the implementation of Gradient Boosting, allowing them to have complete control over the process. By incorporating different regularizations into the loss function, the issue of overfitting that often arises in Gradient Boosting algorithms can be effectively tackled.

XGBoost leverages a blend of classification and regression trees (CARTs) to effectively handle tasks that involve both classification and regression [15]. The prediction model for XGBoost is given using

(1) $${\hat{y}}_{i}=\sum_{k=1}^{K}f_{k}(x_{i}).$$

In Equation (1), ${\hat{y}}_{i}$ denotes the prediction result, *k* is the number of decision trees, and $f_{k}$ represents the *k*-th decision tree.

Initialization (with the absence of any decision tree in the model, the prediction result is 0):

(2) $${\hat{y}}_{i}^{\left(0\right)}=0.$$

We add the decision trees to the model as we go.

First, add

(3) $${\hat{y}}_{i}^{\left(1\right)}=f_{1}\left(x_{i}\right)={\hat{y}}_{i}^{\left(0\right)}+f_{1}\left(x_{i}\right).$$

Second, add

(4) $${\hat{y}}_{i}^{\left(2\right)}=f_{1}\left(x_{i}\right)+f_{2}\left(x_{i}\right)={\hat{y}}_{i}^{\left(1\right)}+f_{2}\left(x_{i}\right).$$

*k*-th, add

(5) $${\hat{y}}_{i}^{\left(t\right)}=\sum_{k=1}^{K}f_{k}(x_{i})={\hat{y}}_{i}^{\left(t-1\right)}+f_{t}\left(x_{i}\right).$$

The loss function in XGBoost comprises two components: the training error denoted as ‘*l*’ and the regularization term denoted as Ω:

(6) $$Obj=\sum_{i=1}^{n}l(y_{i},{\hat{y}}_{i}^{\left(t-1\right)})+\sum_{k=1}^{K}\Omega (f_{K}).$$

With the addition of each decision tree in the model, the loss functions undergo a modification. At the introduction of the *t*-th decision tree, the training process for the preceding *t* − 1 decision trees has concluded, resulting in the training error and regularization term of those *t* − 1 decision trees becoming constants. As a result, the expression of the loss function is

(7) $$Obj=\sum_{i=1}^{n}l\left(y_{i},{\hat{y}}_{i}^{\left(t-1\right)}+f_{t}\left(x_{i}\right)\right)+\Omega \left(f_{t}\right)+C.$$

The regularization term can be rewritten as

(8) $$\Omega \left(f_{t}\right)=\gamma T+\frac{1}{2}\lambda \sum_{j=1}^{T}{\omega o}_{j}^{2}.$$

The added regularization term here helps smooth the final learned weights to avoid overfitting.

*T* represents the number of leaf nodes, and ω denotes the score of the leaf nodes. By substituting Equation (8) into Equation (7), we obtain

(9) $$Obj=\sum_{i=1}^{n}l\left(y_{i},{\hat{y}}_{i}^{\left(t-1\right)}+f_{t}\left(x_{i}\right)\right)+\gamma T+\frac{1}{2}\lambda \sum_{j=1}^{T}\omega_{j}^{2}+C.$$

A second-order Taylor expansion on Equation (9) is conducted, and the following assumptions are established:

(10) $$g_{i}={𝜕}_{{\hat{y}}^{\left(t-1\right)}}l\left(y_{i},{\hat{y}}^{\left(t-1\right)}\right),$$

(11) $$h_{i}={𝜕}_{{\hat{y}}^{\left(t-1\right)}}^{2}l(y_{i},{\hat{y}}^{\left(t-1\right)}),$$

(12) $$G_{i}=\sum_{i\in I_{j}}g_{i},$$

and

(13) $$H_{i}=\sum_{i\in I_{j}}h_{i}.$$

The variable $I_{j}$ in Equations (12) and (13) denotes the sample located on the *j*-th leaf node. The expression of the loss function can be formulated as:

(14) $$Obj=\sum_{j=1}^{T}(G_{j}\omega_{j}+{\frac{1}{2}(H_{j}+\lambda )\omega }_{j}^{2})+\gamma T$$

As shown in Equation (14), the loss function can be seen as a quadratic function about $\omega_{j}$, where there exists a specific $\omega_{j}$ that minimizes the loss function:

(15) $$\omega_{j}^{*}=-\frac{G_{j}}{H_{j}+\lambda }.$$

**Figure 1:**
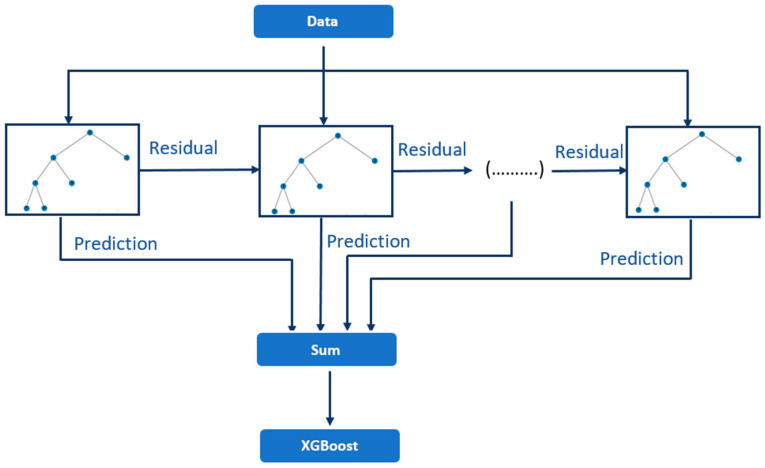
Schematic illustration of XGBoost.

Figure 1 presents a schematic overview of the XGBoost algorithm.

### 3.2. Hyperparameter Tuning in Machine Learning Models

Hyperparameter optimization is a crucial component of machine learning that has a substantial influence on the effectiveness of algorithms. Hyperparameters, in contrast to model parameters, are predetermined before the training process and dictate the global behavior of the model. The approach entails conducting experiments with different combinations of hyperparameters to determine the optimal settings for a given problem [16]. In addition, effective optimization of hyperparameters can result in more precise models by achieving an optimal trade-off between bias and variance, thus mitigating problems such as overfitting or underfitting [17]. In neural networks, hyperparameters such as the number of layers and neurons, learning rate, and activation functions have a direct impact on the model’s capacity to learn complex patterns in the data [18].

The process of fine-tuning the hyperparameters is crucial for optimizing the performance of machine learning models. By adjusting various parameters, such as the learning rate, batch size, or regularization terms, the model can improve its efficiency, resulting in shorter training time and reduced computational resources. This is especially crucial in large-scale applications where resources are limited. In addition, hyperparameter tuning does not have a universal solution.

Various datasets and types of problems may necessitate unique hyperparameter configurations [19]. It is important to note that a high learning rate can result in quick convergence for straightforward problems, but it may introduce instability when training more intricate models. Therefore, mastering the craft of tuning requires a deep comprehension of the data and algorithm dynamics to identify the optimal hyperparameter configurations.

Automated hyperparameter tuning methods such as grid search, random search, and Bayesian optimization have gained significant popularity in recent machine learning practice. These methods thoroughly investigate the hyperparameter space, minimizing the need for manual trial-and-error and enhancing the efficiency of the tuning process while reducing the influence of human biases.

By incorporating these techniques into machine learning workflows, users can shift their attention to other crucial aspects of model development, all while ensuring that their models are performing at their best. In the development of machine learning models, hyperparameter tuning plays a vital role in ensuring their robustness, accuracy, and efficiency [20,21].

### 3.3. Utilization of Metaheuristic Algorithms in Hyperparameter Tuning

#### 3.3.1. Emergence of Metaheuristic Algorithms in Machine Learning

Metaheuristic algorithms have become increasingly important in machine learning, particularly in the field of hyperparameter optimization. These algorithms are exceptionally efficient at dealing with complex hyperparameter spaces, including genetic algorithms, simulated annealing, and particle swarm optimization. They differentiate themselves from traditional approaches such as grid search or random search by not following a pre-established pattern of exploration.

On the other hand, metaheuristic algorithms make use of strategies inspired by natural or biological processes. This innovative method enables them to effectively navigate the search landscape and avoid getting stuck in local optima, which is a common problem. Their remarkable capacity to dynamically navigate through extensive parameter spaces makes them highly efficient in machine-learning scenarios with a large number of hyperparameters and intricate interactions [22].

#### 3.3.2. Superiority of Metaheuristic Algorithms in Exploration

The inherent flexibility of metaheuristic algorithms is a significant advantage in the field of machine learning. Contrary to conventional approaches, metaheuristics take a more dynamic and non-linear approach when exploring the hyperparameter space rather than following a linear or exhaustive search pattern. This enables a comprehensive and intricate examination of possible solutions.

Genetic algorithms, for example, mimic the mechanisms of natural selection and genetic crossover to produce solutions that improve over time. Simulated annealing imitates the process of heating and gradually cooling a material, investigating solutions in a manner that resembles thermal fluctuations.

Particle swarm optimization is inspired by the social behavior of birds flocking, allowing for a collective exploration of the solution space through the movement and interaction of individual ‘particles’ [23].

#### 3.3.3. Enhancing Machine Learning Model Performance

Using varied and inventive exploration strategies, metaheuristic algorithms can uncover hyperparameter combinations that might be missed by conventional approaches. This capability is especially valuable for optimizing complex models, where finding the correct combination of hyperparameters can greatly enhance model performance and prediction accuracy.

The pursuit of optimal or near-optimal solutions not only improves the model’s predictive abilities but also helps to minimize the time and computational resources needed for model training and validation. With the ever-evolving landscape of machine learning, the significance of metaheuristic algorithms in hyperparameter tuning cannot be overstated. These algorithms provide a way to enhance the sophistication and capabilities of machine learning solutions [24].

### 3.4. Genetic Algorithms

Genetic algorithms (GA) are a class of search and optimization algorithms inspired by the process of natural selection and genetics. GA operates on the principle of natural selection, crossover, and mutation to solve complex optimization problems. Genetic algorithms are widely used in various fields, such as engineering, computer science, computational biology, and artificial intelligence.

The fundamental idea behind GAs is to simulate the evolutionary process of biological populations in a digital environment. Concretely, a genetic algorithm begins by representing potential solutions to a given problem as chromosomes or gene sequences, often called individuals or genotypes [25]. These solutions are then evaluated based on their fitness or performance relative to a predefined criterion, usually defined using an objective function.

The most effective individuals are favored for reproduction, while the less effective ones are eliminated or subjected to variation operators such as crossover and mutation. Crossover involves combining the characteristics of two parents to produce offspring, while mutation introduces random changes in genotypes to diversify the population and avoid premature convergence to a local optimum. This process of selection, crossover, and mutation is repeated over several generations until a satisfactory solution or an approximation to the optimal solution is reached [26].

Genetic algorithms provide an efficient heuristic approach for exploring the solution space in complex optimization problems where traditional methods may be ineffective. Their ability to manage large search spaces and find high-quality solutions makes them a powerful tool for solving various engineering, design, and optimization problems in many application areas.

**Figure 2:**
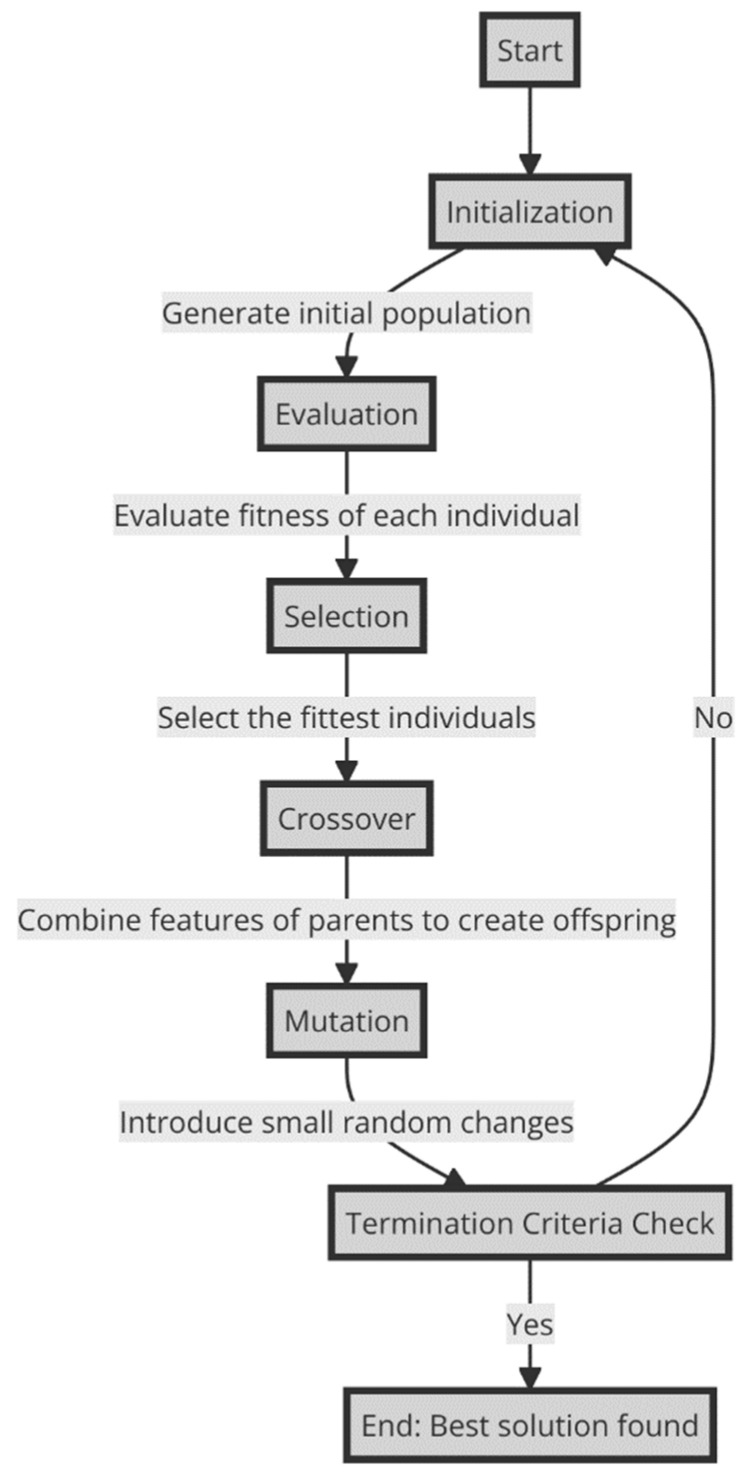
Genetic algorithms process.

In summary, genetic algorithms provide a flexible and scalable framework for solving difficult optimization problems by drawing on the principles of biological evolution. The genetic algorithms can be presented in the Figure 2.

**Figure 3:**
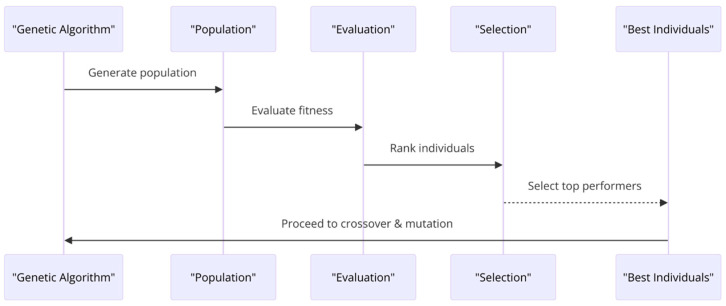
Selection mechanism for genetic algorithms.

**Figure 4:**
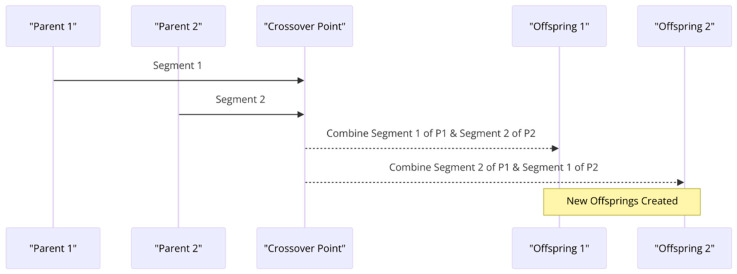
Crossover mechanism for genetic algorithms.

**Figure 5:**
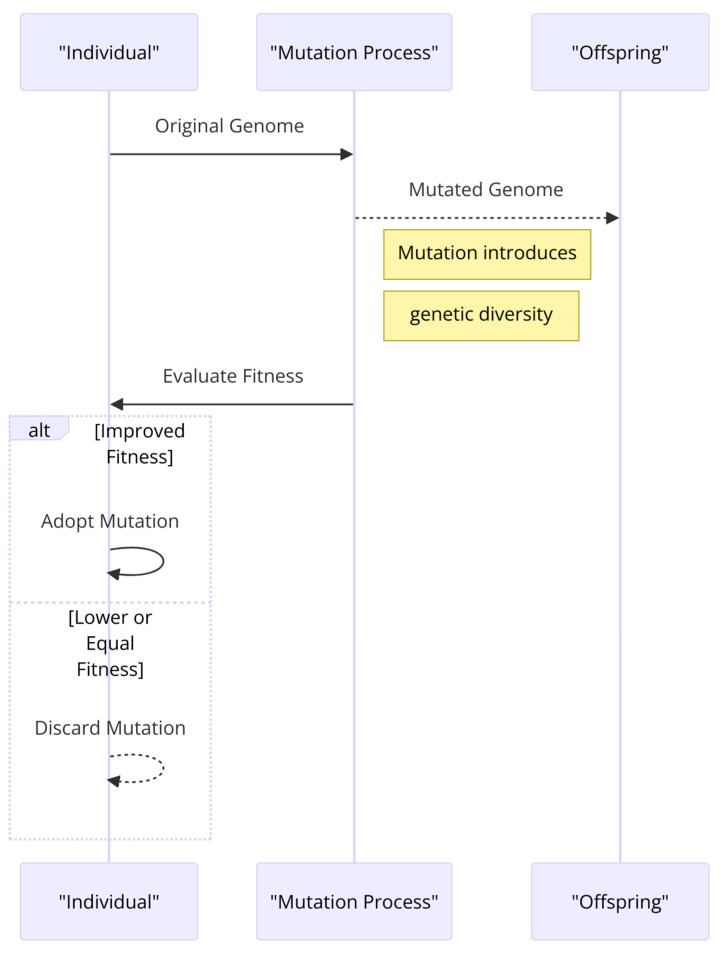
Mutation mechanism for genetic algorithms.

These algorithms are based on several key components, which can be cited as follows:

- Start: The beginning of the genetic algorithm process.
- Initialization: A population of potential solutions is generated. These solutions are usually represented as strings of binary numbers, but they can also be other data structures.
- Evaluation: The objective function, represented by f(x) = y, evaluates the performance of individuals about the defined criterion. Each member of the population is evaluated based on a fitness function. The fitness function determines how close a given solution is to the optimum solution of the problem being solved.
- Selection: This step selects individuals or solutions based on their fitness. The better the solutions, the more chances they have to be selected for reproduction. Selection operators based on techniques such as roulette wheel selection, tournament selection, or rank selection are commonly used to determine the probability of selection of individuals for reproduction. The selection mechanism for genetic algorithms is illustrated in Figure 3.

- Crossover: Also known as recombination, this step combines the genetic information of two parents to generate new offspring. It aims to produce a better solution by combining the best traits of the parents. Figure 4 illustrates the crossover mechanism that is necessary for genetic algorithms.

- Mutation: This step introduces random changes to the offspring’s genetic makeup to maintain genetic diversity within the population, helping to prevent the algorithm from becoming too homogeneous and getting stuck in local optima. Figure 5 illustrates the mutation mechanism that is used in genetic algorithms.

- Termination Criteria Check: This step determines whether the algorithm has met its termination criteria, which could be a specific number of generations, a plateau in fitness levels, or a satisfactory fitness level.
- End: If the termination criteria are met, the algorithm ends, and the best solution found is returned.
- The crossover and mutation-selection operators are the variation operators that promote genetic diversity. Finally, a convergence criterion based on iterations or a definitive fitness value can determine the end of the algorithm [27]. These combined components allow genetic algorithms to efficiently explore the solution space and converge on optimal solutions in a variety of optimization problems.

### 3.5. Application and Efficacy of Genetic Algorithms in XGBoost Hyperparameter Search

When it comes to hyperparameter tuning for models like XGBoost, one approach that stands out is the use of genetic algorithms (GAs). XGBoost is highly regarded for its exceptional performance in various machine learning tasks. It relies on several crucial hyperparameters, including the learning rate, max depth, and number of trees. These hyperparameters have a significant impact on the overall performance of XGBoost.

The optimization of these parameters is achieved through a process that closely resembles biological evolution. It utilizes mechanisms such as selection, crossover, and mutation. This method enables a flexible and responsive investigation of the hyperparameter space.

Recent studies comparing genetic algorithms with more conventional hyperparameter tuning methods have revealed that GAs cannot only match but, in certain instances, surpass the performance of these standard approaches. In addition, these results are often achieved more efficiently, which is especially valuable considering the computational intensity of training models such as XGBoost [28].

The impressive achievements of genetic algorithms in these studies underscore the immense possibilities of employing advanced metaheuristic methods to enhance the optimization of machine learning models. Implementing these methods can result in notable enhancements in model accuracy and efficiency, ultimately improving the overall effectiveness of machine-learning applications [29].

**Figure 6:**
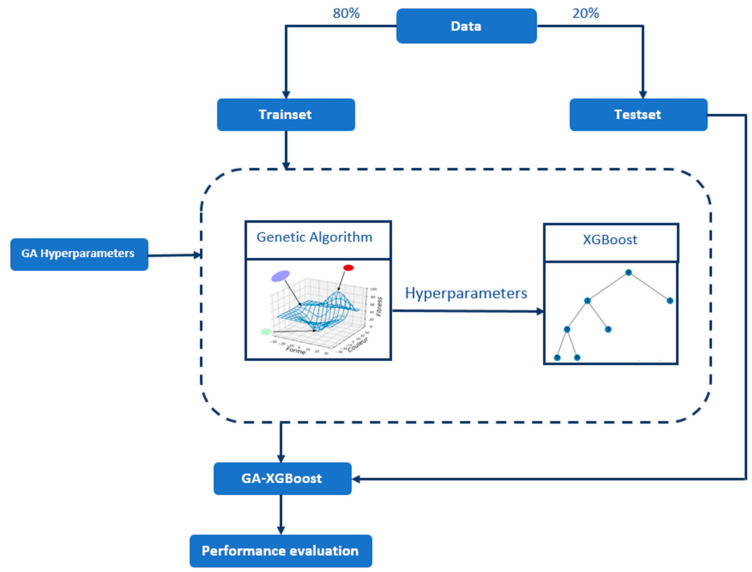
The Combined Model of GA-XGBoost.

The application of metaheuristic algorithms, particularly genetic algorithms, in hyperparameter tuning, showcases a notable progression in the realm of machine learning. Using advanced optimization techniques, professionals can achieve superior model performance, leading to more precise and effective predictive models. Figure 6 illustrates the combined model of the GA-XGBoost algorithm.

### 3.6. Objective Functions and Solution Encoding/Decoding

This section delves into enhancing the XGBoost model’s performance via Genetic Algorithms (GAs) for hyperparameter optimization. It outlines our methodology for encoding and decoding hyperparameter values into genetic representations, facilitating efficient exploration of the hyperparameter space, and surpassing traditional optimization methods like grid or random search [30,31].

**Figure 7:**
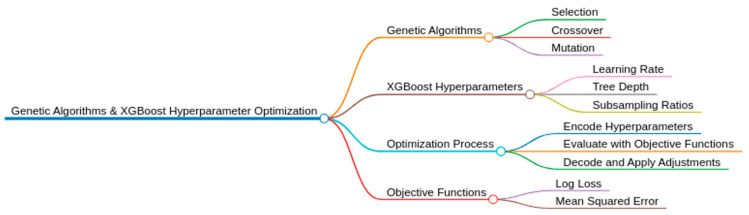
Improved XGBoost based on genetic algorithm.

In our approach, each set of XGBoost hyperparameters is represented as an individual within the GA population, encoded into chromosomes consisting of genes. Each gene corresponds to a specific hyperparameter value, such as the learning rate or number of trees, enabling a structured and efficient optimization process. Figure 7 illustrates an improved version of the XGBoost algorithm, which incorporates genetic techniques.

#### 3.6.1. Encoding Solutions

Our GA utilizes a mixed encoding strategy to accommodate the diverse nature of XGBoost’s hyperparameters, encompassing discrete, continuous, and categorical types:

Binary Encoding: Numeric hyperparameters are converted into binary strings, with the string’s length reflecting the desired precision. For instance, the learning_rate might be represented using binary string encoding values within the 0.01 to 0.3 range.

Real-valued Encoding: This method directly uses the hyperparameter values as chromosomes, suitable for parameters like n_estimators and max_depth, mapped to their respective ranges.

String Character Conversion: Categorical hyperparameters, such as objective, are encoded using unique string or character representations, allowing for the effective inclusion of categorical variables in the optimization process.

#### 3.6.2. Decoding Solutions

The decoding process translates the GA-encoded representations back to the specific XGBoost hyperparameters. This translation is straightforward for real-valued and string character encodings, while binary encodings require conversion from binary strings to decimal values mapped to the hyperparameter range.

#### 3.6.3. Genetic Operations

Chromosomes are the genetic representation of a full set of XGBoost hyperparameters.

Mutations introduce variation by randomly altering genes, aiding in exploring the hyperparameter space.

Crossover combines genes from two parent chromosomes, generating offspring that inherit a mix of hyperparameters, facilitating the discovery of new and potentially more effective solutions.

#### 3.6.4. Genetic Algorithm Variants

Our analysis considers several GA variants, each suitable for different aspects of hyperparameter optimization:

Simple Genetic Algorithm (SGA): Utilizes binary encoding, ideal for discrete hyperparameters.

Real-coded Genetic Algorithm (RGA): Preferred for continuous hyperparameters, directly operates with real values, enabling finer adjustments within the search space.

Genetic Programming (GP): Although not directly applied to hyperparameter optimization, GP strategies inspire innovative approaches to handling structured hyperparameters.

Given the complexity and high-dimensional nature of the hyperparameter space in XGBoost models, the Real-coded Genetic Algorithm (RGA) emerges as particularly suitable for our dataset. Its capability to handle continuous hyperparameters directly facilitates more nuanced adjustments, potentially leading to quicker convergence on optimal or near-optimal solutions, especially for datasets with a mix of categorical and numerical features.

## 4. SGCC Dataset and Exploration

The dataset provided by the State Grid Corporation of China (SGCC) is a valuable resource for conducting energy research and analysis. This dataset presents a statistical analysis of electricity consumption over 1036 days, including data from 42,372 customers. This extensive dataset is valuable for various energy management studies, smart grid development, and sustainable practices. It provides valuable insights into consumer behavior, energy utilization patterns, and the effectiveness of grid management strategies. The description of the SGCC Dataset is presented in Table 2.

The SGCC dataset will likely contain comprehensive records of electricity usage, carefully organized by residential, commercial, and industrial sectors and covering various periods. This data is crucial for understanding consumption patterns comprehensively and plays a vital role in forecasting demand. This particular aspect of the dataset is of utmost importance when it comes to analyzing energy usage trends and devising effective strategies to meet future energy demands.

This dataset is anticipated to offer comprehensive insights into electricity distribution across different regions, encompassing crucial information on grid capacities, transmission efficiencies, and load management. This information is crucial for studies focused on grid optimization and ensuring the reliability of energy supply.

Gaining insights into the distribution and management of energy in various regions can result in notable enhancements in the efficiency and stability of power grids. The SGCC dataset provides comprehensive data on the physical infrastructure of energy supply, including substations, transmission lines, and maintenance records. These data are crucial for infrastructure planning and development studies, enabling informed decisions on upgrades, expansions, and maintenance of energy supply systems.

A crucial aspect of the dataset pertains to incorporating sustainable energy sources into the power grid. It provides generation statistics and efficiency metrics for renewable energy, offering valuable insights for those studying renewable energy integration and sustainability. These data are essential for gaining insights into the impact and effectiveness of renewable energy sources in today’s energy systems.

The dataset likely includes data on energy conservation programs, efficiency improvements, and demand response efforts. These aspects play a crucial role in energy policy and efficiency optimization studies, contributing to the development of effective energy conservation and sustainable energy conservation strategies.

The extensive scope of the SGCC dataset makes it an invaluable resource for those involved in research and policy-making. It has a wide range of applications, such as analyzing and predicting electricity consumption trends, planning and optimizing grid infrastructure and distribution networks, developing renewable energy integration strategies, and evaluating energy efficiency and conservation policies. This dataset serves as a valuable resource for various research areas, making significant contributions to the progress of energy management and sustainable practices.

**Figure 8:**
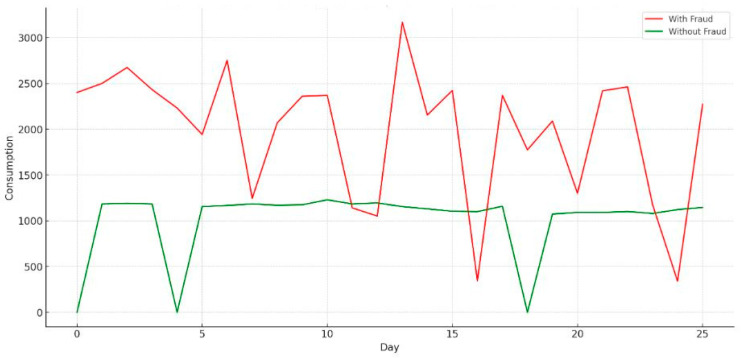
Comparison of electricity consumption: With fraud vs. without fraud.

Overall, the SGCC dataset’s extensive scope and comprehensive nature provide a distinct chance for interdisciplinary exploration in the energy field, making notable contributions to the progress of energy management, sustainability, and smart grid technologies. Figure 8 presents a comparison of the electricity consumption in the presence and absence of fraud.

### 4.1. Data Pre-Processing

In addressing the challenge of missing data within the Smart Grid Consumption Dataset (SGCC), an interpolation method is employed to ensure data integrity and continuity, which is crucial for accurate consumption analysis and fraud detection. Interpolation, a statistical technique used to estimate missing values based on the available data, assumes that the change between points is smooth over the dataset’s range. This method is particularly beneficial in time-series data, like the SGCC, where consumption readings are sequential and closely related. The interpolation method is shown in Equation (16):

(16) $$f\left(x_{i}\right)=\left\{\begin{array}{c} \frac{x_{i+1}+x_{i-1}}{2}\;if\;x_{i}\in NaN,\;x_{i+1}\;and\;x_{i-1}\;\notin NaN\;\\ \;0\;if\;x_{i}\in NaN,\;x_{i+1}\;or\;x_{i-1}\;\in NaN\;\\ \;x_{i}\;if\;x_{i}\notin NaN\; \end{array}\right.,$$

where $x_{i}$ is the feature of the electricity consumption data and *NaN* means a missing value. In addition, we incorporated the Min–Max scaling method to normalize the data within the range of 0 to 1 [8,32]. Data normalization enhances the training process of deep learning models by ensuring a consistent scale across the dataset. The following equation is employed for data normalization:

(17) $$A^{\prime }=\frac{A-Min(A)}{Max\left(A\right)-min(A)\prime }.$$

Here, Maj$A^{\prime }$ represents the normalized value. The effectiveness of machine learning algorithms depends on the quality of input data. Data pre-processing serves to enhance both the quality of data and the performance of these models.

### 4.2. Dataset Balancing in SGCC for Energy Theft Detection

Balancing datasets (SGCC) for detecting energy theft is essential. This is because the occurrence of energy theft is usually much less frequent compared to legitimate energy usage, creating an imbalance in the data. This imbalance can severely impact the effectiveness of machine learning models, making them prone to overlook theft instances by favoring the more common legitimate usage data. To counter this, various methods are used to balance the data, ensuring that the models can accurately detect instances of theft:

1. Oversampling the Minority Class: This method increases the number of theft cases in the dataset by either duplicating existing cases or creating new, synthetic examples. A popular technique for generating these synthetic examples is SMOTE (Synthetic Minority Over-sampling Technique), which creates new instances by blending between actual examples [33].
2. Undersampling the Majority Class: Conversely, this approach reduces the number of legitimate usage instances to equal the theft cases, though it may result in the loss of some valuable data [34].
3. Cost-sensitive Learning: This strategy modifies the learning algorithm to impose heavier penalties for misclassifying theft cases than for incorrect identifications of legitimate usage, focusing the model more on the minority class [35].
4. Ensemble Methods: These methods use multiple models to improve detection accuracy. Techniques like Balanced Random Forest and EasyEnsemble are designed to balance the dataset implicitly during model training [36].

For SGCC applications, where detecting energy theft is paramount, combining SMOTE with ensemble methods is highly effective. This approach not only retains the valuable information in the majority class but also ensures sensitivity toward detecting theft instances. The use of ensemble methods further amplifies detection accuracy by leveraging the strengths of multiple models.

**Figure 9:**
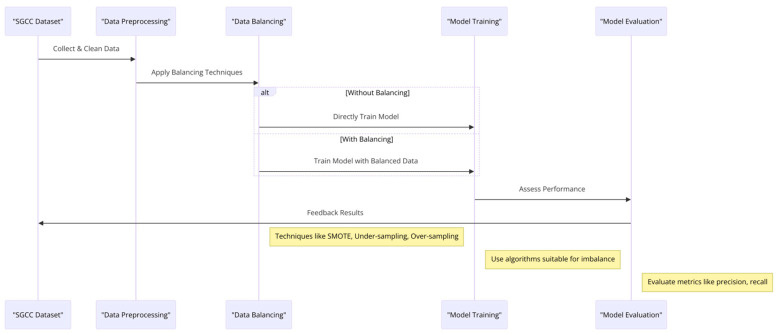
Machine learning workflow for imbalanced dataset handling.

Implementing SMOTE involves generating new samples by adjusting the difference between a minority class sample and its nearest neighbors with a random factor. For ensemble methods like the Balanced Random Forest, multiple decision trees are trained on balanced subsets of the dataset, enhancing the model’s ability to identify theft accurately. Figure 9 illustrates the workflow for handling imbalanced datasets in machine learning.

The subdivision of datasets into training, validation, and test sets constitutes a pivotal phase in crafting robust machine-learning models, with a particular emphasis on the reproducibility of outcomes. This procedure, commonly referred to as data splitting, underpins the integrity of model performance evaluation.

The essence of data splitting lies in its provision of a truthful appraisal of a model’s efficacy. By allocating distinct datasets for the training and testing phases, it is ensured that the model’s performance is gauged on data it has not previously encountered, thereby simulating real-world application conditions. This aspect is of paramount importance in applications involving models such as XGBoost for tasks like theft detection, where inaccuracies in prediction carry significant repercussions. Moreover, consistency in data splitting is crucial for enabling reproducibility, facilitating the replication of experiments, and verification of results by other researchers. This practice ensures that observed enhancements in model performance are attributed to authentic algorithmic improvements rather than overfitting specific datasets.

In our examination, the State Grid Corporation of China (SGCC) dataset was segmented into three discrete subsets: training, validation, and test sets. The training set serves as the foundation for the XGBoost algorithm to discern the data’s inherent patterns. The validation set is utilized for the fine-tuning of hyperparameters through Genetic Algorithms (GAs), aiming to forestall overfitting throughout the learning process.

Conversely, the test set, unseen by the model during training, is employed to assess the final model’s performance. This segmentation was executed employing stratified sampling to preserve the class distribution inherent to the original dataset, a step of critical importance for imbalanced datasets such as the SGCC. Stratified sampling guarantees that each segment accurately reflects the dataset’s full class spectrum, including the minority class, thereby maintaining the data’s characteristics and ensuring a reliable evaluation of the model’s capabilities in theft detection [37].

The training phase involves the employment of the XGBoost algorithm, enabling the model to learn from the training set’s patterns. Concurrently, the validation phase incorporates Genetic Algorithms for the optimization of hyperparameters, a measure designed to prevent the model from overfitting during its learning trajectory.

The final evaluation of the model’s performance is conducted using the test set, which is critical for ensuring the model’s ability to operate effectively on unseen data. This step is especially crucial for applications such as theft detection, where the cost of false predictions can be substantial.

An efficacious strategy for data division involves the application of a stratified split, which upholds the class proportions uniformly across both training and test datasets, thus mirroring the comprehensive dataset composition. This division ought to be executed with an unchanging random state, a designated integer seed, facilitating the exact replication of data segmentation in subsequent analyses. Utilizing the train_test_split function from Scikit-learn’s model selection module, with a specified random_state and stratify argument, achieves this objective.

Further enhancing the robustness of evaluation metrics, the incorporation of k-fold cross-validation, anchored by a consistent random state throughout the model’s training phase, diminishes variance through the employment of varied data subsets. The diligent application of these methodologies, coupled with the meticulous documentation of the specific parameters engaged for data splitting and cross-validation, guarantees the precision of the experimental framework. This meticulous approach ensures the experimental rigor and authenticity of the theft detection model’s evaluative process, reinforcing its scientific validity.

The meticulous division of data into training, validation, and test sets is indispensable for the development of machine-learning models that are both robust and reproducible. This study underscores the significance of data splitting, not only as a means of ensuring model reliability and performance but also as a cornerstone for the reproducibility of scientific findings in the machine learning domain.

## 5. Results and Discussion

### 5.1. Performance Evaluation Metrics

Machine learning models undergo performance evaluation using various metrics, including Precision, Recall, F1 Score, and Accuracy. These metrics play a crucial role in assessing a model’s effectiveness across different scenarios. They are derived from the confusion matrix, a fundamental tool generated during a model’s testing phase. This matrix furnishes the necessary information to compute Precision, Recall, F1 Score, and Accuracy. Each of these metrics is determined using specific mathematical formulas, often referenced as equations below in relevant literature. These computations aid in gauging the model’s capacity to make accurate predictions and its ability to distinguish between different classes.

- True Positive (TP): Dishonest consumers correctly identified as dishonest.
- True Negative (TN): Honest consumers correctly identified as honest.
- False Positive (FP): Honest consumers mistakenly identified as dishonest.
- False Negative (FN): Dishonest consumers mistakenly identified as honest.

Precision: This measure assesses the accuracy of positive predictions by determining the fraction of correctly identified positive instances. It holds particular significance in situations where the consequence of a false positive is significant. Precision is computed by dividing the number of true positives by the sum of true positives and false positives.

Recall (Sensitivity or True Positive Rate): Recall evaluates the model’s capacity to detect pertinent instances accurately. It holds particular significance in scenarios where failing to identify a positive example (false negative) can have significant repercussions. Recall is computed by dividing the number of true positives by the sum of true positives and false negatives.

F1 Score: The F1 Score is a statistical metric representing the harmonic means of Precision and Recall. This measure is well-balanced because it considers both false positives and false negatives. It proves especially valuable in situations where the distribution of classes is imbalanced, as it offers a more reliable assessment of performance compared to relying solely on accuracy.

Accuracy: Accuracy is a key performance metric in machine learning, defined as the ratio of correct predictions to the total number of predictions made using a model. This metric is particularly reliable and straightforward when the dataset has a balanced class distribution and when the impact of false positives and false negatives is approximately equal. In such scenarios, accuracy provides a clear and intuitive assessment of a model’s predictive performance.

Confusion Matrix: This matrix is a vital evaluation tool in machine learning for assessing classification model performance. It visually maps a model’s predictions against actual outcomes, categorizing them into four key segments: True Positives (TP), True Negatives (TN), False Positives (FP), and False Negatives (FN). This classification aids in determining not only the model’s overall accuracy but also specific error types, such as overpredictions (FP) and underpredictions (FN).

The matrix is foundational in computing other critical performance metrics like precision, recall, F1-score, and accuracy. Precision calculates the ratio of correctly predicted positive observations, whereas recall measures the ability to identify actual positives correctly. The F1-score, a harmonic mean of precision and recall, is especially valuable in scenarios with imbalanced class distributions. Accuracy offers a general sense of the model’s correctness across both positive and negative predictions.

Mastering the Confusion Matrix is crucial for machine learning practitioners, as it significantly influences the efficiency and dependability of predictive models. Together, these metrics offer a detailed view of a model’s performance, highlighting strengths and pinpointing weaknesses, particularly in managing different classification error types.

### 5.2. Results and Optimization

XGBoost, a powerful and widely used machine learning algorithm, offers a myriad of hyperparameters that can be fine-tuned to enhance model performance. Employing genetic algorithms for hyperparameter tuning presents a robust approach to navigating the vast parameter space of XGBoost. The following table (i.e., Table 3) delineates some of the crucial hyperparameters of XGBoost, offering a brief definition of each and suggesting intervals for exploration in the tuning process:

The application of the Genetic Algorithm (GA) to optimize the XGBoost model’s hyperparameters significantly enhanced its performance in detecting consumer fraud within the SGCC dataset. The hyperparameters adjusted through GA included learning_rate, n_estimators, max_depth, min_child_weight, gamma, subsample, colsample_bytree, reg_alpha, reg_lambda, scale_pos_weight, and objective.

Table 4 shows a comparison of these hyperparameters before and after optimization. Every parameter was meticulously adjusted, leading to a model that is more suitable for the unique characteristics of the SGCC fraud detection task.

### 5.3. Model Performance

**Figure 10:**
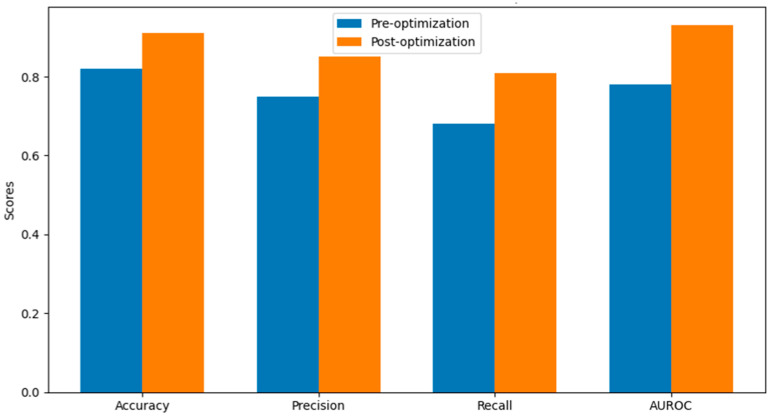
Model performance metrics Pre- vs. Post-optimization.

The impact of hyperparameter optimization on the model’s performance was evaluated using accuracy, precision, recall, and AUROC (as shown in Table 5). Figure 10 depicts these metrics, illustrating substantial improvements, particularly in recall and AUROC, post-optimization.

### 5.4. Discussion

The results underscore the effectiveness of using GA for fine-tuning hyperparameters in an XGBoost model designed for detecting fraudulent activities. The remarkable enhancements in recall and AUROC after optimization are especially impressive, demonstrating a strong ability to detect fraudulent activities, which is essential in real-world situations.

The adjustments made to hyperparameters like max_depth, subsample, and scale_pos_weight were crucial. The significance of fine-tuning the model to address the intricacies and imbalances present in the SGCC dataset is highlighted using these modifications. The transition from binary:logistic to binary:logitraw in the objective function further customized the model to better suit the unique requirements of the fraud detection task.

Although the findings show promise, it is crucial to consider the potential variability caused by the stochastic nature of GA. Additional investigation may delve into the consistency of these findings across various iterations and datasets. In addition, exploring the model’s performance on data from different periods or demographics within the SGCC dataset could provide more profound insights into its relevance and efficiency.

Our findings align closely with prior research on hyperparameter optimization techniques and their application in fraud detection, demonstrating a strong synergy. Notably, several papers [8,9,12] have emphasized the significance of advanced machine learning algorithms in improving the precision and effectiveness of fraud detection systems. Our research takes the foundations a step further using Genetic Algorithms to fine-tune hyperparameters in XGBoost models, resulting in a significant improvement in detection performance metrics. The remarkable increase in model accuracy from 0.82 to 0.978, along with improvements in precision, recall, and AUROC, is consistent with the positive results reported in [12] when utilizing hybrid CNN-Transformer models. This analysis serves to strengthen the reliability of our methodology and emphasizes the significance of our study in the field of smart grid fraud detection.

## 6. Conclusions

The integration of digital technologies into electrical grids is resulting in increased connectivity, enhanced reliability, and improved sustainability. Numerous endeavors have been undertaken to revitalize the electric grid and update systems that have been in existence for over a century. It is anticipated that in the foreseeable future, a significant transformation in power grid monitoring will occur, yielding remarkable outcomes by leveraging artificial intelligence, big data, drones, and other emerging technologies [38,39,40].

This study successfully demonstrates the power of integrating Genetic Algorithms (GA) with the XGBoost model for hyperparameter optimization in the context of fraud detection using the SGCC dataset. The research reveals that such an approach significantly enhances model performance, particularly in terms of accuracy, precision, recall, and AUROC. This advancement is crucial in the field of electricity theft detection, where accurately identifying fraudulent activities is paramount.

The modifications in key hyperparameters like learning_rate, n_estimators, max_depth, min_child_weight, gamma, subsample, colsample_bytree, reg_alpha, reg_lambda, scale_pos_weight, and objective underline the importance of a nuanced approach towards hyperparameter tuning, especially in complex and imbalanced datasets like the SGCC. The notable improvement in accuracy from 0.82 to 0.978 post-optimization highlights the effectiveness of GA in navigating the hyperparameter space of XGBoost more efficiently compared to traditional methods.

In addition, this research paves the way for future exploration, especially in utilizing similar methodologies with various datasets and contexts. This study emphasizes the importance of ongoing innovation in algorithmic approaches to address the changing challenges in fraud detection. It suggests integrating advanced machine learning techniques in practical applications such as electricity theft detection.

This work highlights a notable advancement in improving the precision and effectiveness of fraud detection systems in smart grids, providing a hopeful method for guaranteeing more secure and dependable power distribution networks. This research not only contributes significantly to the domain of fraud detection in smart grids but also opens avenues for further exploration in the application of advanced metaheuristic algorithms for optimizing machine learning models. The findings encourage continued advancements in this field, aiming to enhance such models’ accuracy, efficiency, and applicability in real-world scenarios.

In conclusion, integrating GA with XGBoost presents a promising and effective approach for tackling the challenges in electricity theft detection, paving the way for more secure and reliable power distribution systems.

In addition, future studies could prioritize real-time application and the ability to explain the model. Enhancing the model’s implementation in real-time systems could greatly improve its practical impact and user trust by enabling immediate fraud detection and enhancing its explainability. Further investigation into advanced techniques for tuning hyperparameters and adapting to changing fraud tactics would help maintain the model’s relevance and effectiveness in the long run. These viewpoints underscore the ever-changing nature of machine learning applications, emphasizing the importance of ongoing innovation and adaptation.

## Figures and Tables

**Table 1:** Overview of the relevant work related to electricity theft detection.

Paper Reference	Methodology	Focal Point	Key Findings	Implications	Results	Future Perspectives
Khan et al., 2020, [8]	Data preprocessing, Adasyn for class balancing, VGG-16 for feature extraction, FA-XGBoost for classification.	ETD using smart meter data.	Improved classification with VGG-16 in ETD.	Applicable for identifying electricity theft and reducing wastage.	High F1-score, precision, and recall values.	Further refinement of ML models for real-world application in varied electricity grids.
Aslam et al., 2020, [9]	LSTM–UNet–Adaboost with IQMOT for class balancing.	Electricity theft detection using smart meters.	High detection rate, surpassing traditional methods.	An efficient solution for real-time ETD.	Achieved a detection rate of 0.92.	Exploration of additional deep learning architectures for more robust theft detection.
Saglam et al., 2022, [10]	ANN, PSO, and MLR for electricity demand forecasting.	Forecasting electricity demand for Gokceada Island.	ANNs showed a strong correlation with actual data.	Assists in managing electricity demand growth.	High confidence interval for the ANN method.	Application of forecasting models to other regions and integration with renewable energy sources.
Badawi et al., 2022, [11]	Two-stage process using ARIMA and DRF.	Detection of fraudulent NTL in smart grids.	Achieved 98% accuracy and F1-score in NTL detection.	A robust method for detecting electricity fraud in smart grids.	Outperformed state-of-the-art methods.	Enhancement of fraud detection algorithms to adapt to evolving theft techniques.
Bai et al., 2023 [12]	Hybrid CNN-Transformer model (DSDBGWT) for feature extraction.	Electricity theft detection in smart grids.	Enhanced efficiency in feature extraction.	A comprehensive approach for effectively detecting electricity theft.	High F1 scores and AUC values, indicating notable robustness.	Optimization of the model for faster processing and broader adaptability in different grid systems.
Khattak et al., 2022, [13]	Hybrid DL model combining GRU and CNN, with ADASYN and TomekLinks for class imbalance.	Electricity theft detection using smart meter data.	High PR-AUC and ROC-AUC, outperforming benchmarks.	Effective in detecting electricity theft and improving accuracy in smart grids.	Achieved 0.985 PR-AUC and 0.987 ROC-AUC.	I am investigating scalability and efficiency improvements, and application to diverse datasets.

**Table 2:** SGCC Dataset description.

Attribute Description	Values
Period of data collection	2014–2016
Data type	Time series
Dimension	1034
Samples	42,372
Number of fraudulent consumers	3800
Number of honest consumers	38,530
Total consumers	42,372

**Table 3:** XGBoost hyperparameters intervals.

Hyperparameter	Definition	Search Interval
learning_rate (eta)	The rate at which the model adapts to new data. Controls overfitting.	0.01 to 0.3
n_estimators	The number of boosting rounds or trees to build.	50 to 1000
max_depth	The maximum depth of each tree. Higher values make the model more complex and prone to overfitting.	3 to 10
min_child_weight	The minimum sum of instance weight (hessian) needed in a child node.	1 to 10
gamma (min_split_loss)	Minimum loss reduction is required to make a further partition on a leaf node. Increases model conservatism.	0 to 5
subsample	The fraction of samples to be used for training each tree. Aids in preventing overfitting.	0.5 to 1
colsample_bytree	The fraction of features (columns) used per tree. Reduces overfitting.	0.3 to 1
reg_alpha	L1 regularization term on weights. Higher values lead to more regularization.	0 to 1
reg_lambda	L2 regularization term on weights. More effective in highly imbalanced cases.	1 to 100
scale_pos_weight	A value to balance class distribution. Useful for imbalanced datasets.	10, 15, 20
objective	Specifies the learning task and the corresponding learning objective.	binary: logistic binary: logitraw

**Table 4:** XGBoost hyperparameters before and after optimization.

Hyperparameter	Pre-Optimization	Post-Optimization
learning_rate	0.1	0.07
n_estimators	100	450
max_depth	6	10
min_child_weight	1	5
gamma	0	0.4
subsample	1.0	0.8
colsample_bytree	1.0	0.7
reg_alpha	0	0.3
reg_lambda	1	0.8
scale_pos_weight	1	20
objective	binary: logistic	binary:logitraw

**Table 5:** Model performance before and after optimization.

Metric	Pre-Optimization	Post-Optimization
Accuracy	0.82	0.978
Precision	0.75	0.92
Recall	0.68	0.89
AUROC	0.78	0.96

## Data Availability

Data is contained within the article.
